# Towards Rebalancing Blood Pressure Instability after Spinal Cord Injury with Spinal Cord Electrical Stimulation: A Mini Review and Critique of the Evolving Literature

**DOI:** 10.1016/j.autneu.2021.102905

**Published:** 2021-11-11

**Authors:** Madeleine Burns, Ryan Solinsky

**Affiliations:** a Boston University School of Medicine, Graduate Medical Sciences; b Spaulding Rehabilitation Hospital; c Department of Physical Medicine & Rehabilitation, Harvard Medical School; d Spaulding Research Institute

## Introduction

Beyond motor paralysis, chronic spinal cord injury (SCI) results in a diverse range of comorbidities and effects multiple organ systems. Not least among these are problems with blood pressure instability. This may present acutely in individuals with high-level lesions as orthostatic hypotension (OH, where uncompensated blood pressure acutely falls in response to a postural challenge) and autonomic dysreflexia (AD, where blood pressure rises to potentially dangerous levels from below level sympathetic activation). This unbalanced blood pressure control, with fluctuating pressures, is thought to contribute to the significantly accelerated rate of vascular disease in individuals with cervical SCIs; their risk of heart disease and stroke being three- and four-fold higher, respectively, compared to uninjured peers (*Myers et al.*, 2007, *Phillips et al.*, 2015). From a spinal cord physiology standpoint, regulation of the cardiovascular system is controlled primarily through pathways exiting the thoracic spine (*Landrum et al.*, 1998, *Teasell et al.*, 2000). Interruption in descending signals due to SCI results in cardiovascular dysregulation, manifesting as instability in blood pressure control (*Landrum et al.*, 1998, *Teasell et al.*, 2000). In one moment, an individual with chronic SCI may present with profound hypotension (OH), to the point of losing consciousness, while the next they may display severe, episodic bouts of life-threatening hypertension (AD, *Claydon et al.*, 2006, *Harkema et al.*, 2018a).

Normally, cardiovascular control originates in the brainstem and passes through the bulbospinal tract of spinal cord, terminating at sympathetic preganglionic neurons in the imtermediolateral cell column. These cells are influenced by a myriad of interneurons (*Deuchars et al.,* 2011) before sending signals to act on the periphery. With SCI, there are varying degrees of interruption of this descending bulbospinal central sympathetic control (*Cormier et al*., 2010). Hence, therapeutic strategies that target normalization of descending spinal control pathways may be of great value in restoring blood pressure instability for these individuals.

The application of electrical stimulation to the spinal cord represents an emerging approach with a growing number of provocative early results for management OH and AD in individuals with SCI. Differing from typical pharmacological approaches, this electrical stimulation aims to provide a locally targeted effect below the injury level. Moreover, this stimulation provides an important window to uncover the physiological infrastructure and plasticity of the human spinal circuitry. Developing a deeper understanding of how the neural circuitry that controls the cardiovascular system can be manipulated with spinal cord electrical stimulation after SCI is crucial to clinical implementation of this technology.

## Cardiovascular Dysregulation following Spinal Cord Injury

**Figure 1:**
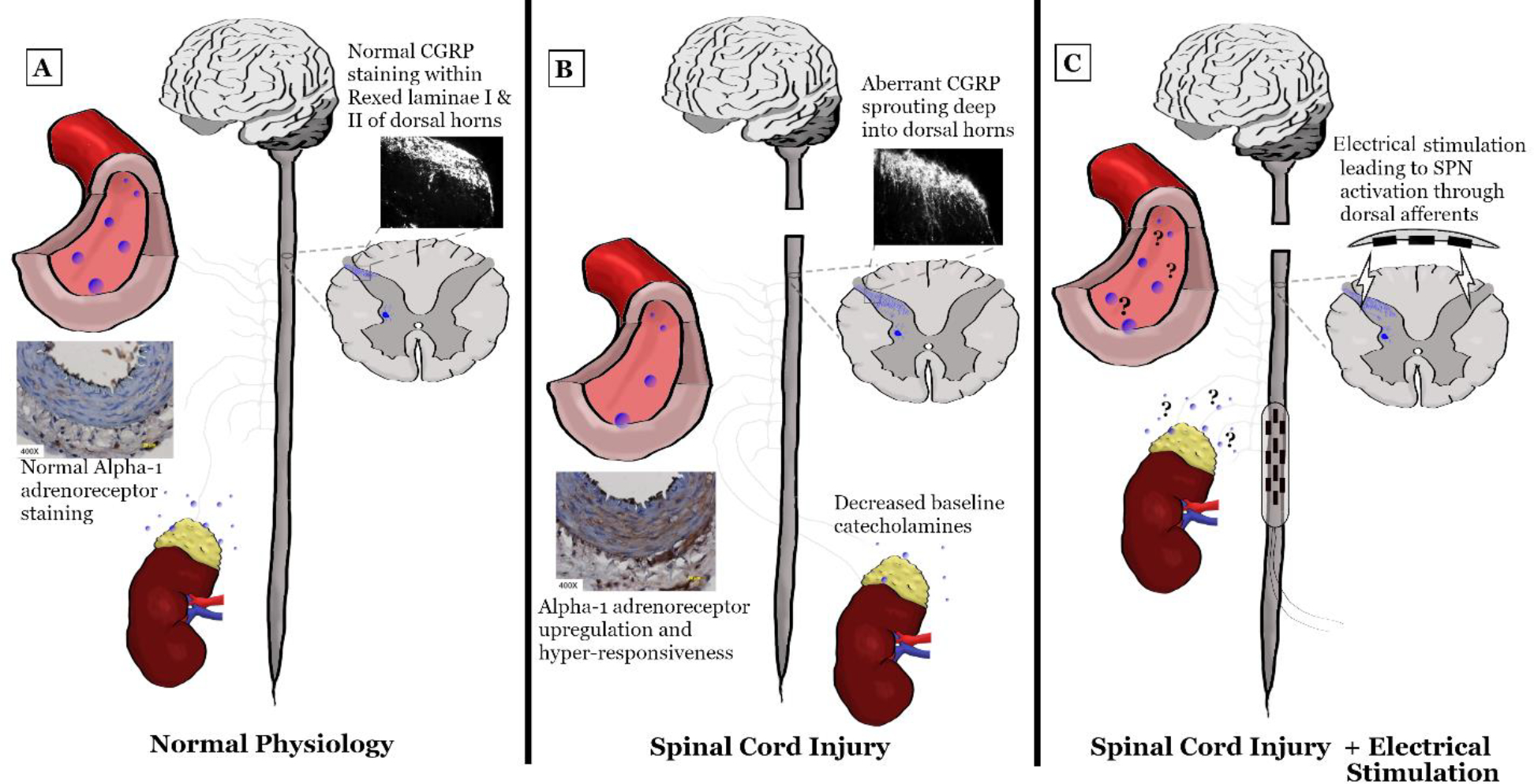
Proposed response of vessels and adrenal medulla in an uninjured individual (A), an individual with SCI (B) and an individual with SCI undergoing spinal cord electrical stimulation (C, proposed mechanism). Of note, alpha-1 adrenoreceptor staining consistent with vessel hyper-responsiveness is increased following SCI (Panel B, *Lee et al.*, 2016), though its changes are unknown after spinal cord electrical stimulation. Baseline norepinephrine levels and impairment in adrenal responsiveness is also classically seen after SCI (Panel B, *Claydon et al*., 2006, *Mathias et al.*, 1975, *Wecht et al.*, 2018), though it is unknown how spinal cord electrical stimulation may affect this. Calcitonin gene related peptide (CGRP) reactive afferents are known to sprout within the dorsal horns after SCI, leading to amplification of sympathetic responsiveness (*Krenz et al.,* 1998). Though again, remains unknown if spinal cord electrical stimulation modifies these branched dendritic arbors. SPN = sympathetic preganglionic neurons.

In the setting of impaired descending bulbospinal sympathetic control, SCI commonly results in a host of secondary autonomic adaptations (*Phillips et al.*, 2015). While full review of these changes is beyond our scope, and have been well summarized elsewhere (*Eldahan et al*., 2017, *Phillips et al.*, 2015, *Teasell et al.*, 2000), briefly both reduction of basal sympathetic tone below the injury (as seen in OH with chronically low norepinephrine levels and impaired adrenal responsiveness, Figure 1, *Claydon et al*., 2006, *Mathias et al.*, 1975, *Wecht et al.*, 2018) and hyperresponsivity during sympathetic reflex engagement (as seen in AD) classically occur (*Phillips et al.*, 2015). Additionally, acutely after SCI, an outpouring of local nerve growth factor in the setting of inflammation, leads to sprouting of sensory afferent fibers in the dorsal horns, forming new connections (*Krenz et al.*, 1998, *Brennan et al*., 2021, *Mironets et al*., 2020, *Squair et al*., 2021). These connections can variably influence sympathetic preganglionic neurons, likely through intermediary sympathetic interneurons (*Schramm*, 2006). Finally, with low resting catecholamine levels in individuals with cervical SCI, alpha-adrenoreceptor hyperresponsiveness also occurs and accentuates the pressor response specifically in AD (Figure 1, *Rodriguez et al.*, 1986, *Teasell et al.*, 2000).

These host of changes in cardiovascular autonomic control after SCI can manifest as both OH (where systolic blood pressure falls at least 20 mmHg when orthostatically challenged, *Wecht et al*. 2018) and AD (where systolic blood pressure increases over 20 mmHg from baseline, commonly due to overdistension of the bladder, *Krassioukov et al*. 2012, *Solinsky et al*., 2016). While clinically significant and associated with negative long-term health consequences, both OH and AD are under-recognized as they often lack accompanying symptoms (*Mathias et al.*, 1975, *Linsenmeyer et al*., 1996, *Kirshblum et al.*, 2002, *West et al.,* 2016, *Juraschek et al*., 2018). Utilizing ambulatory intermittent blood pressure monitoring, OH has been estimated to occur over 9 times per day on average, while individuals at risk for AD may experience an average of 13 episodes per day (*Hubli et al.*, 2015, *Dance et al.*, 2017). The recurrent nature of these drastic blood pressure fluctuations from OH and AD leads to increased endothelial shear stress and has been postulated to contribute to the high risk for cardiovascular disease in this population (*West et al.*, 2016, *Aslan et al.*, 2018, *Currie et al*., 2019). Compounding this issue, pharmacological interventions that are used to manage blood pressure instability often fall short because of undesirable side effects and the need for advanced planning to treat the unpredictable episodes of orthostatic or hypertensive stress (*Wecht et al*., 2018, *Thyberg et al*., 1994). As a result, those with chronic SCI have few options to maintain adequate systemic blood pressure. For this reason, investigation of effective treatment options for blood pressure instability following SCI is of paramount importance.

## Spinal Cord Electrical Stimulation

While the autonomic dysregulation that results secondary to SCI is crucial to address, many recent, high profile studies have explored epidural spinal cord stimulation to regain volitional movement after paralysis. In a growing number of individuals, these studies have shown the ability of targeted spinal cord electrical stimulation to restore movement, standing, and walking in individuals with clinically complete paralysis (*Harkema et al.*, 2011, *Angeli et al.*, 2014, *Angeli et al.*, 2018, *Gill et al.*, 2018, *Wagner et al.*, 2018). As these studies geared towards motor recovery progress, they provide further insights into the underlying mechanism of spinal cord stimulation which can be leveraged to understand its potential applications in the autonomic nervous system.

Computational models suggest that epidural electrical stimulation sends bidirectional action potentials through the lumbosacral spinal cord, engaging nascent circuits to facilitate movement (*Darrow et al.*, 2019, *Milosevic et al.*, 2019). The stimulation thereby recruits large-diameter dorsal root afferent circuits to activate interneurons and motoneurons, increasing the overall excitability of the spinal cord and promoting the integration of load-bearing proprioceptive inputs and coordination of motor activity (*Capogrosso et al.*, 2018, *Formento et al.*, 2018, *Jack et al.*, 2020). Similarly, transcutaneous spinal cord stimulation has been shown to cause local excitatory effects within the spinal cord of individuals with SCI (*Benavides et al*., 2020). More recent literature has pointed to the benefits of spatially selective and temporally specific bursts of epidural electrical stimulation to engage segregated motor pools through individual dorsal roots. This phase-dependent regulation of proprioceptive feedback circuits steers the stimulation-derived excitation toward leg motor neuron pools that directly align with a stage of movement with good specificity (*Wagner et al*., 2018, *Capogrosso et al.*, 2018). During such motor-based explorations of epidural stimulation, it was incidentally noted that blood pressure also transiently increased with the stimulator on (*Harkema et al*., 2018a). This prompted investigators to explore the potential use of this technology for cardiovascular control in these individuals with SCI. While there is still much to learn regarding how spinal cord electrical stimulation controls locomotion, this evidence provides the scientific community with neurophysiological markers to select from when determining stimulation parameters and lays important groundwork of core physiology for autonomic neuromodulation (*Milosevic et al.*, 2019).

## Using Spinal Cord Electrical Stimulation to Target the Cardiovascular System

Spinal cord electrical stimulation has shown promise for mitigating cardiovascular dysregulation within the SCI population. Specifically, West *et al.* demonstrated that epidural stimulation caused a well-controlled rise in blood pressure in one individual and prevented orthostatic hypotension (Table 1, *West et al.*, 2018). In this case, the stimulation counteracted the decrease in end diastolic volume during a tilt table challenge, likely by inducing vasoconstriction below the neurological level of injury (*West et al.*, 2018). Additionally, these authors suggest that epidural stimulation in individuals with SCI did not cause AD, noting that stimulation maintains a controlled low grade basal sympathetic tone, and keeps blood pressure elevated while not triggering episodes of extreme hypertension (*West et al.*, 2018). Such naturally occurring basal sympathetic tone has recently been identified in individuals with SCI, though the stimulus amplitude administered in epidural stimulation may need to be continually tuned to prevent additional uncontrolled cascades of sympathetic activation (*Solinsky et al.*, 2019). Further emerging research on cardiovascular-targeted epidural stimulation corroborates these findings. Evidence by *Darrow et al.* demonstrates that epidural stimulation administered to two individuals with chronic SCI triggers immediate clinical relief of OH (Table 1, *Darrow et al.*, 2019). Likewise, Harkema and colleagues found that stimulation induced significant and reproducible increases in blood pressure in four individuals with SCI, resolving their symptomatic hypotension (Table 1, *Harkema et al.*, 2018a). *Phillips et al*. reproduced these blood pressure increases using transcutaneous spinal cord stimulation in five individuals with SCI and further demonstrated increases in cerebral artery flow velocities (Table 1, *Phillips et al.* 2018). In all these studies, the authors claim these increases in blood pressure are the result of improved autonomic neuroregulation, though notably, only one study applied epidural stimulation without postural challenge (Table 1, *Harkema et al.*, 2018a).

In the most recent and comprehensive study to date, *Squair et al*. utilized both preclinical rodent and non-human primate animal models and a single human participant with SCI to assess epidural stimulation’s ability to regulate blood pressure and prevent hypotension (Table 1, *Squair et al.*, 2021). In the rodent SCI model, they fist mapped spinal cord stimulation locations from T6-L1 and then identified that the strongest pressor response coincided with the localized highest density of sympathetic preganglionic neurons innervating the splanchnic vasculature (T11-T13 in their rodent model). They further identified the large diameter fibers within the spinal posterior roots as the key modulatory entry point into the spinal cord, with progressive root ablation leading to a suppressed pressor response. Drawing from sympathetic nerve recordings, they then developed a biomimetic closed-loop epidural stimulation system to adaptively apply stimulation based on continual blood pressure targets. This stimulation strategy was repeated in non-human primates with SCI, normalizing sympathetic nerve activity and resting catecholamine levels. Following this, a single human participant with complete C6 SCI and OH was implanted with this device. Epidural stimulation led to resolution of his OH without requiring any additional pharmacologic management.

In a comparative study of individuals with chronic SCI with and without baseline hypotension, Aslan and colleagues found that only the hypotensive group (consequently with low basal levels of catecholamines) demonstrated an increase in blood pressure with epidural stimulation (Table 1, *Aslan et al.*, 2018). The authors postulate that the lack of pressor response in normotensive individuals with SCI suggests that epidural stimulation is improving autonomic neuroregulation, effectively not impacting those where this regulation is already intact (*Aslan et al.*, 2018). However, it is also possible that the electrical stimulation causes a low-grade AD in the individuals with baseline OH, whereas those without OH (and presumably a more intact autonomic regulatory system in their spinal cord) are less susceptible to this induced AD. This potential criticism has been raised previously when similar claims have been made (*Minic et al*., 2018). Lastly, findings from *Legg Ditterline et al.* recently revealed the prophylactic potential of prolonged, daily epidural stimulation (Table 1, *Legg Ditterline et al.*, 2021). In their study, these authors established that active electrical stimulation during postural stress may not be necessary following prolonged exposure, as chronic, recurrent stimulation prevented OH from arising even without the stimulator turned on (Table 1, *Legg Ditterline et al.*, 2021). The underlying autonomic mechanisms of this are still unknown, and further well-controlled studies are needed to ensure that utilizing epidural stimulation for OH does not lead to increased magnitude or frequency of AD through creation of aberrant sympathetic plasticity within the spinal cord.

While there is growing evidence for spinal cord stimulation mitigating OH following SCI, far less has been reported on the role of spinal cord stimulation to prevent or attenuate AD. In animal SCI studies, transcutaneous spinal electrical stimulation attenuated the severity of hypertension and completely resolved the AD caused by induced colonic distension (Table 1, *Collins et al.*, 2002). These findings were corroborated by a human case series of five individuals with SCI who frequently presented with episodic AD (Table 1, *Richardson et al.*, 1979). In this study, it was found that constant, daily neurostimulation every two to three hours for at least eighteen months completely eliminated symptoms of AD in four of five individuals with SCI for as long as a year after completion (*Richardson et al.*, 1979). However, if the stimulation was not tapered gradually, the individual would immediately exhibit symptoms characteristic of AD, suggesting that the autonomic neuroregulation may be dependent on the chronicity and consistency of stimulation (*Richardson et al.*, 1979). Recently, *Sachdeva et al*. reproduced these animal results with transcutaneous spinal cord stimulation with biphasic pulses, demonstrating both prevention of AD from colonic distension and the ability of electoral stimulation to nullify AD which was occurring (Table 1, *Sachdeva et al*., 2021). These authors further tested transcutaneous stimulation on a single human participant with C4 complete SCI, mirroring their results from the animal study. Overall, the studies exploring spinal cord electrical stimulation for clinical prevention or mitigation of AD are far less robust than those for OH. Understanding the mechanisms for how this stimulation specifically acts to improve blood pressure control will be crucial to its clinical implementation.

## Proposed Theoretical Mechanisms for Addressing Blood Pressure Instability with Spinal Cord Electrical Stimulation

While spinal cord electrical stimulation is an active area of current research, the mechanisms of action require further rigorous scientific dissection. To date, several theories have been proposed to explain the clinical findings. For OH, one prominent theory is that epidural stimulation activates dorsal afferent relays, causing an increase in the resting membrane potential of sympathetic circuits in the lumbosacral spinal cord (*Harkema et al.*, 2018a, *West et al.*, 2018). Ultimately, this theory suggests the increase in sympathetic tone results in vasoconstriction of the peripheral arteries and splanchnic vascular bed, leading to an increase in venous return and a rise in blood pressure (*Harkema et al.*, 2018a). However, as an emerging field, other alternative mechanisms for electrical stimulation’s modulation of blood pressure have been postulated. *Legg Ditterline et al.* suggest a mechanism that relies on feed-forward vasopressor effects, namely increased baroreflex sensitivity which they deduce from changes in spectral analysis of heart rate variability (*Legg Ditterline et al.*, 2021). They postulate this relates to “increased stimulation of baroreceptors in response to systolic blood pressure changes, which led to significant decreases in heart rate during orthostatic stress” (*Legg Ditterline et al.*, 2021). Another study postulates that stimulation results in a “bionic reflex,” where intact baroreceptors in the aortic arch and carotid sinus are activated by epidural stimulation, causing a decrease in heart rate but also increasing vascular tone in order to re-stabilize the cardiovascular system (*Harkema et al.*, 2018a).

Mechanistically, these regulatory theories on how lumbosacral electrical stimulation acts on distant cardiovagal baroreceptors are difficult to physiologically rationalize. More likely, sympathetic induced vasoconstriction results in increased pressure which is buffered by baroreflex mediated bradycardia and shift in the observed spectral densities. That these studies both analyze spontaneous indices without paced breathing further introduces a confounder, as electrical stimulation following SCI is known to alter pulmonary function, which may change interfering respiratory frequency bands (*Dimarco et al.*, 2019). Although these hypotheses speak to the baroreflex’s ability to generate vagally mediated bradycardia, they neglect the fact that individuals with SCIs commonly lack the ability to regulate sympathetic outflow below the level of injury. If spinal cord electrical stimulation was reactivating this spinal efferent arm of the baroreflex, originating in the medulla, baroreceptor activation leading to the observed bradycardia would be driven by systemic pressure being interpreted as too high, causing the medulla to decrease sympathetic firing, not increase it to cause vasoconstriction. While these mechanistic theories have all been put forth, further study, as highlighted below, is needed to more fully understand these stimulation paradigms.

Just as the clinical literature on spinal cord electrical stimulation for AD management lags behind that of OH, the proposed theories for mechanism are also earlier in development. The proposed mechanisms for how electrical stimulation could alleviate AD further contrast with those of OH by the nature of activating inhibitory circuits rather than excitatory sympathetic relays. One theory introduced by *Richardson et al.* is that the stimulatory device creates a synthetic central inhibitory mechanism between the intact “decerebralized” spinal cord and the peripheral elements, therefore preventing episodes of hyperactivation of the sympathetic system, as seen in AD (*Richardson et al.*, 1979). These authors postulated that epidural stimulation elicits net sympathetic inhibition or parasympathetic facilitation - essentially bypassing previous afferent sensory networks that may have triggered AD (*Richardson et al.*, 1979). On the other hand, *Collins et al.* propose that electrical stimulation decreases the firing of dorsal horn neurons, preventing their consequent activation of the sympathetic vasoconstrictor response, which is characteristic of AD (*Collins et al.*, 2002). Mechanistically, how electrical stimulation can active these inhibitory pathways (for AD) while also engaging sympathetic networks (for OH) raises questions about this theory. *Sachdeva et al*. propose that despite similar stimulation parameters, the biphasic aspect of spinal stimulation preferentially activates inhibitory interneurons, though this has yet to be tested. Finally, one historical physiologic reflex, the Lovén reflex, which induces skeletal muscle vasodilation in response to lumbar dorsal root electrical stimulation may also have a potential role, thought this has yet to be explored (*Lovén*, 1866, *Jänig* 2021). With the presence of multiple competing proposed mechanisms to explain the clinical findings, well-informed and focused autonomic research will assuredly be required.

Given the incongruities with these mechanistic theories, understanding the underlying mechanism of action of spinal stimulation on the cardiovascular system is a crucially important next step. This will likely require additional preclinical animal models with shared endpoints with human participants with SCI, as multiple recent studies have done (*Squair et al*., 2021, *Sachdeva et al*., 2021).

## Evidence of Studies to Date

### Novel Risks Associated with Spinal Cord Electrical Stimulation

Differing from systemic pharmacologic agents, spinal cord electrical stimulation may require implanted stimulator leads (epidural stimulation) or superficial stimulation through the skin (transcutaneous stimulation), both of which have associated risks. Epidural stimulators used for pain mitigation in individuals without SCI are known to carry risk of epidural hematoma, occurring in 0.2–0.8% of procedures (*Bendersky et al*., 2014, *Petraglia et al*., 2016). These adverse events are typically identified by patients experiencing new neurological symptoms or focal pain exacerbation. In individuals with pre-existing SCI and associated sensory loss, it is unknown how epidural hematoma would present. Given that transcutaneous spinal cord stimulation is non-invasive, adverse events are typically limited to skin irritation after SCI (*Sayenko et al*., 2019). However, as individuals with SCI are commonly at high risk for even minor appearing skin irritation to develop into a clinically significant pressure injury (*Lemmer et al*., 2019), caution is warranted.

## Future Study

Spinal cord electrical stimulation holds promise for the SCI community, however, there is still much to be learned given the variability of the pathophysiology after these injuries. Because of this variability, studies to date have utilized nonstandard stimulation sites and parameters, custom to each individual. Current studies demonstrate that stimulation used to treat hypertension and attenuate the sympathetic response seen in AD can be introduced at a multitude of levels of the thoracic spinal cord (Table 1). In contrast, stimulation for OH generally targets the T10-S2 spinal cord in most studies (Table 1). Ultimately, future studies must confirm the specific common sites of spinal cord stimulation to treat the unpredictable shifts from AD to OH in an individual patient, thereby refining protocols for advancing research and wider clinical implementation of this technology (*Solinsky et al.*, 2020b).

To answer the pending mechanistic questions on spinal cord electrical stimulation for OH, future studies should pair stimulation with catecholamine levels in both orthostatically challenged and unchallenged positions. If spinal cord electrical stimulation in the supine position leads to increases in norepinephrine and systolic pressure increase of > 20 mmHg, this would be indicative that it is inducing AD and not improving some unmeasured regulatory pathway. Researchers should specifically address the effects of prolonged spinal cord electrical stimulation lasting hours as opposed to just a single minutes long bout, as prolonged stimulation will likely be needed clinically. Further, following this chronic spinal cord electrical stimulation, it will be important to measure thresholds for induction of AD, to ensure that in artificially increasing blood pressure to prevent OH, this stimulation does not swing the pendulum so treated individuals have more frequent or higher magnitude dysreflexia (*Harkema et al*., 2018b).

The path forward for spinal cord electrical stimulation to treat and prevent AD is less well defined. Future studies are needed in basic animal models as well as multi-participant human cohorts to more robustly corroborate animal data. Further, the optimal stimulation target (dorsal horns, dorsal root ganglia, sympathetic preganglionic neurons) needs to be identified, as this may dictate which device/electrodes will need to be utilized (*Parker et al*., 2021, *Guiho et al*., 2021). Given the highly skewed clinical etiology of AD toward noxious causes originating in the bladder, these studies should focus on controlled, bladder-induced dysreflexia through urodynamics. Common spinal stimulation sites, shared with spinal cord electrical stimulation for OH, would maximize eventual clinical translation of these findings, as most individuals with SCI who experience AD also have episodes of OH.

Additionally, while studies to date have focused on blood pressure changes with spinal cord electrical stimulation, the cardiovascular autonomic regulatory system is obviously more complex than this. Changes in compensatory cardiovagal baroreflex sensitivity should be analyzed as well as the spectral analysis phase relationships between stimulation induced shifts in blood pressure and heart rate responses. As these phase relationships have been shown to be reversed following SCI (with feedback mechanism of blood pressure into heart rate), rectification could signal more natural cardiovascular autonomic regulation (*Solinsky et al.*, 2020a).

## Summary

Although questions regarding mechanism and optimal stimulation persist, evidence indicates that spinal cord electrical stimulation has potential to be an effective treatment of OH and may have a role in AD management in individuals with SCI. Future preclinical studies aimed at identifying key underlying mechanisms for AD and OH, as well as larger human studies to demonstrate clinical applicability in “real world” settings will explicate many of the questions that remain surrounding the configurations for effective stimulation, ultimately rebalancing blood pressure instability following SCI.

## Figures and Tables

**Table 1:** Summary of studies to date exploring epidural stimulation for blood pressure instability following spinal cord injury.

**Orthostatic Hypotension management with Spinal Cord Electrical Stimulation**					
***Study Author (date)***	**Number of Subjects/Animal Model**	**Participant Characteristics (human only)**	**Spinal Level of Stimulation, Frequency and Current**	**Measured Outcomes**	**Major Findings**
Aslan et al. (2018)	N=3 hypotensive humans, N=4 normotensive humans	NLI= C5-T4 AIS= A, B Mean TSI= 2.7 years Mean Age= 26.7 years	L1-S1 epidural stimulation, 15–35 Hz, unknown mA	Arterial blood pressure during supine and manually assisted sitting.	Stimulation in three individuals with OH resulted in increased arterial blood pressure. Stimulation in four individuals without OH did not cause an increase in blood pressure.
Darrow et al. (2019)	N=2 humans	NLI= T4, T8 AIS= A Mean TSI= 7.5 years Mean Age= 50 years	L2-S2 epidural stimulation, 16–400 Hz, 2–15 mA	Arterial blood pressure during tilt challenge	Stimulation in one individual with OH resulted in increased blood pressure, while stimulation in another individual without OH did not affect cardiovascular function.
Harkema et al. (2018a)	N=4 humans	NLI= C4 AIS= A, B Mean TSI= 6.5 years Mean Age= 30.8 years	L1-S1 epidural stimulation, 30–60 Hz, unknown mA	Mean arterial blood pressure and heart rate in an upright, seated position.	Stimulation increased mean arterial pressure and decreased or kept heart rate constant.
Legg Ditterline et al. (2021)	N=4 humans † same individuals as Harkema et al. (2018a)	NLI= C4 AIS= A, B Mean TSI= 6.5 years Mean Age= 30.8 years	L1-S1 epidural stimulation, unknown Hz, unknown mA	Blood and heart rate variability, as well as baroreflex function with an orthostatic stress test. Circulating norepinephrine levels.	Stimulation increased blood pressure, heart rate variability and baroreceptor effectiveness. Norepinephrine levels unable to be detected at all time points, with and without stimulation.
Phillips et al. (2018)	N=5 humans	NLI= C5-T2 AIS= A, B Mean TSI= >3 years Mean Age= ? (range 23–32 years)	T8 transcutaneous stimulation, 30 Hz, up to 70 mA	Beat-to-beat blood pressure, heart rate during supine and manually assisted sitting. Blood flow velocity of MCA and PCA. Subjective rating of nausea/dizziness.	Stimulation increased blood pressure, heart rate, and MCA/PCA flow velocity compared nadir of orthostatic challenge. Decreased subjective ratings.
Squair et al. (2021)	N=3 rhesus monkeys N=1 human	NLI= C5 AIS= A TSI= ? Age= 38 years	T10-L1 epidural stimulation, 120 Hz, 0–7.5 mV variable	Beat-to-beat blood pressure, muscle sympathetic nerve activity, supine and in 70º tilt table. Circulating norepinephrine levels.	Blood pressure increased in proportion to calibrated stimulation. Reported increases in sympathetic nerve activity on microneurography and norepinephrine levels.
West et al. (2018)	N=1 human	NLI= C5 AIS= B TSI= ? Age= “early 30s”	T10-L2 epidural stimulation, 35 Hz constant, unknown mA	Beat-to-beat blood pressure, cardiac function in a supine position and then in response to a 60º head-up tilt.	Stimulation increased blood pressure and resolved OH.
**Autonomic Dysreflexia management with Spinal Cord Electrical Stimulation**					
***Study Author (date)***	**Number of Subjects/Animal Model**	**Participant Characteristics (humans only)**	**Spinal Level of Stimulation, Frequency and Current**	**Measured Outcomes**	**Major Findings**
Collins et al. (2002)	N=11 rats	NA	T12-S3 TENS stimulation, 60 Hz, 600 μA	Blood pressure response to graded colon distension triggering AD with and without stimulation.	Attenuated the hemodynamic response to colon distension and decreased the change in arterial blood pressure.
Richardson et al. (1979)	N=5 humans	NLI*= ? AIS*= ? Mean TSI= 2.0 years Mean Age= 20.6 years	T12-L3 epidural stimulation, 7–200 Hz, 0.1–14V	Clinical findings only.	Case studies of five individuals with AD. Stimulation prevented further episodes of AD.
*Sachdeva et al*. (2021)	N= 43 rats N=1 human	NLI= C4 AIS= A TSI= 3 years Age= 37 years	T7–8 transcutaneous stimulation, 30 Hz, 20–30 mA (biphasic pulses)	Beat-to-beat blood pressure, heart rate during digital anorectal stimulation.	Prevention of AD and decrease in systolic pressure with stimulation compared to control.

NLI = neurological level of injury. AIS = American Spinal Injury Impairment Scale. TSI = time since injury. OH = orthostatic hypotension. MCA = middle cerebral artery. PCA = posterior cerebral artery. AD = autonomic dysreflexia. TENS = transcutaneous electrical nerve stimulation.

* Based upon clinical data presented, predates AIS.
